# Evaluation of stemness marker expression in bovine ovarian granulosa cells

**DOI:** 10.21451/1984-3143-AR2018-0083

**Published:** 2019-10-23

**Authors:** Son Nghia Hoang, Chi Nguyen Quynh Ho, Thao Thi Phuong Nguyen, Chung Chinh Doan, Diem Hong Tran, Long Thanh Le

**Affiliations:** 1 Animal Biotechnology Department, Institute of Tropical Biology, Vietnam Academy of Science and Technology, Ho Chi Minh City, Vietnam.; 2 Department of Internal Medicine - Cardiology, UT Southwestern Medical Center, Dallas, TX.

**Keywords:** granulosa cells, ovarian follicles, transcript expression, stemness markers

## Abstract

The objective of this study was to assess the stemness marker expressions (Oct4, Nanog, and Sox2) of granulosa cells (GCs) collected from bovine ovarian follicles and *in vitro* expansion. The single bovine ovarian follicles were isolated and categorized into 4 groups according to their diameter including group A (<2 mm), group B (2-3 mm), group C (3-4 mm), and group D (>4 mm). Quantitative reverse transcriptase polymerase chain reaction (qRT-PCR) and immunostaining were applied to evaluate the stemness marker expression of bovine GCs from ovarian follicles. We also estimated the stemness marker transcript expressions of GCs during *in vitro* expression by qRT-PCR. qRT-PCR analysis demonstrated that fresh GCs from bovine ovarian follicles expressed the stemness markers (Oct4, Nanog, Sox2). These markers were down-regulated during antral stage follicular development. We also estimated stemness marker transcript expressions of GCs which were isolated and *in vitro* expanded from ovarian follicles of group A. The qRT-PCR results showed that Oct4 and Sox2 transcript expressions were reduced during *in vitro* expansion while Nanog transcript was not expressed.

## Introduction

The mammalian ovarian follicle development is begun from the primordial follicle, which contains an immature small egg and a single undifferentiated granulosa cell layer (Gougeon, 1996). This cell layer is surrounded by a follicular wall (Gougeon, 1996). During follicular development, the surrounding GCs begin to undergo the process of maturation, the number of GCs increases and the basal membrane is enlarged (Rodgers *et al*., 1999; 2001). The rapid proliferation of GCs suggests that there is an involvement of stem cell based-mechanisms during folliculogenesis (Chronowska, 2012). Many studies have provided evidence that GCs carry the characteristics of stem cells (Chronowska, 2012). Stem cell properties can be displayed in GCs by lacking mitotic inhibition of transcription (Rodgers *et al*., 1999), the ability to divide without anchorage to a substratum *in vitro* (Potten *et al*., 1990; Copper, 1997). Following the study of Kossowska-Tomaszczuk on luteinizing GCs isolated from the ovarian follicles of infertile patients, OCT4, a typical stem cell marker, was expressed in long-term culture with the presence of the leukemia-inhibiting factor (Kossowska-Tomaszczuk *et al*., 2009).

The potential properties of GCs have been characterized in human (Kossowska-Tomaszczuk *et al*., 2009; Parte *et al*., 2011 ; Varras *et al*., 2012), mouse (Bagheripour *et al*., 2017), porcine (Mattiolia *et al*., 2012; Oki *et al*., 2012). These studies characterized the stemness marker expression of GCs in developing follicles from specific stages. An evidence for ovarian granulosa stem cells in bovine follicles was firstly demonstrated with high activity of telomerase (Lavranos *et al*., 1999). However, the potential of bovine ovarian GCs has not been well described, especially the changes of stemness marker expression in follicular development. Thus, the current study was aimed to investigate the expression and changes of stemness markers (Oct4, Nanog, Sox2) in bovine GCs from developing follicles. The results of this study could contribute to understanding of potential of bovine GCs.

## Materials and Methods

### Isolation and culture bovine granulosa cells

Bovine ovaries were obtained from slaughter house and transported to the laboratory within 3 hours. They were kept in 0.9% normal saline. Single bovine follicles were collected by dissection under a stereomicroscope (Meiji, Japan).

To estimate the stemness transcript expression of fresh GCs, four bovine follicle groups were selected based on diameter including group A (< 2 mm), group B (2-3 mm), group C (3-4 mm), and group D (> 4mm). Single bovine follicles were washed in Dulbecco’s Modified Eagle Medium (DMEM, Gibco, Germany) supplemented with 10% fetal bovine serum (FBS, Gibco, Germany) and 1% antibiotic (penicillin/streptomycin, Gibco, Germany). The fresh GCs were collected from these above follicles by aspiration and the oocyte-cumulus complexes were removed. These fresh GCs were used for stemness transcript analysis by qRT-PCR.

To estimate the stemness transcript expression of GCs from *in vitro* expansion, the granulosa from group A were collected and cultured. Bovine GCs were aspirated and transferred to Φ 35 mm tissue culture dishes containing DMEM supplemented with 10% FBS and 1% penicillin/streptomycin. The oocyte-cumulus complexes were removed from collecting GCs. GCs were cultured at 37.5^o^C in a 5% CO_2_ atmosphere. The GCs from passage 0, passage 1, and passage 4 were used for stemness transcript analysis by qRT-PCR. In this examination, the GCs of passage 0 were different from fresh GCs. They were isolated from follicle and primarily cultured for several days. After exhibiting 90% confluence, the primary cultured GCs of passage 0 were collected and used for transcript expression analysis.

### Total RNA extraction

The bovine GCs were harvested and washed twice with phosphate-buffered saline (PBS) (Gibco, Germany). Their total RNA was extracted using a Ribospin™ Total RNA Purification Kit (GeneAll Biotechnology, Korea), according to the manufacturer’s instructions. The quality and quantity of the RNA sample were assessed using the spectrophotometer (NanoVue Plus Spectrophotometer, GE Healthcare Life Sciences, United States). RNA samples with a ratio of A_260_/A_280_ between 1.8 and 2.0 were aliquoted and used for qRT-PCR.

### Quantitative Real time RT-PCR

PikoReal 96 Real-Time PCR System (Thermo Scientific, United States) was used for qRT-PCR. qRT-PCR reactions were performed with qPCR SyGreen 1-Step Lo-ROX kit (Biosystem, England). qRT-PCR was conducted in 20 μl for each reaction, including 1 μl of total RNA, 2 μl of primers (forward and reverse) (Table 1) (Sumer *et al*., 2011), 10 μl Mix Ro-Lox, 1 μl RTAse, and 6 μl dH_2_O. The qRT-PCR reactions were performed by one cycle of 45°C for 15 min, one cycle of 95°C for 2 min, 40 cycles of 95°C for 10 sec, 62°C for 15 sec; and 71 cycles of 60°C for 30 sec. β-actin was used as internal control, the 2^-∆∆Ct^ method was applied for Ct value analysis (Livak *et al*., 2001). PCR products were electrophoresed on a 1% agarose gel and stained with GelRed (Biotium, United States). The gel was exposed to UV light and the picture taken with a gel documentation system (Infinity, Vilber Lourmat, France).

**Table 1:** Primers were used in this study for qRT-PCR.

Gene	Primer (5’–3’)	Product size (bp)
Oct4	F: GTTCTCTTTGGAAAGGTGTTC R: ACACTCGGACCACGTCTTTC	313
Nanog	F: GTGTTTGGTGAACTCTCCTG R: GGGAATTGAAATACTTGACAG	307
Sox-2	F: CATCCACAGCAAATGACAGC R: TTTCTGCAAAGCTCCTACCG	251
β-actin	F: GGAATCCTGTGGCATCCATGAAAC R: AAAACGCAGCTCAGTAACAGTCCG	348

### Immunostaining

This assay was applied to assess the OCT4 and NANOG expression from fresh GCs. GCs were collected from bovine ovarian follicle of group A and cultured in Φ 35 mm tissue culture dish containing DMEM supplemented with 10% FBS and 1% penicillin/streptomycin. After 3 h, GCs attached to dishes and were used for OCT4 and NANOG immunostaining assays. GCs were fixed with 4% paraformaldehyde in PBS (Nacalai, Japan) at room temperature for 30 min, then permeabilized with 0.1% Triton X-100 in PBS (Merck, Germany) at room temperature for 30 min. GCs were incubated with diluted primary antibodies overnight at 4°C for OCT4 (sc-8629, Santa Cruz Biotechnology, United States) and NANOG (sc-30328, Santa Cruz Biotechnology, United States). GCs were incubated with donkey anti-goat IgG-FITC (sc-2024, Santa Cruz Biotechnology, United States) at room temperature in the dark for an hour. GC nucleus was stained with 4′,6-Diamidino-2-phenylindole dihydrochloride (DAPI) (Sigma, United States) for 30 min. The cells were washed with PBS three times in 5 min for each step. The stained GCs were observed under the fluorescent microscope to assess the OCT4 and NANOG expression.

### Statistical analysis

Quantitative gene expression results were analyzed by Piko Real Software 2.2 software (Thermo Scientific, United States). β-actin was used as an internal control. Statistical analysis was performed by Sigma Plot 14 Software (Systat Software Inc., UK). The experiments were triplicated. The stemness transcript expressions were analyzed for statistical significance by one-way ANOVA where P < 0.05 was considered statistically significant.

## Results

### Stemness marker expression of fresh GCs

**Figure 1:**
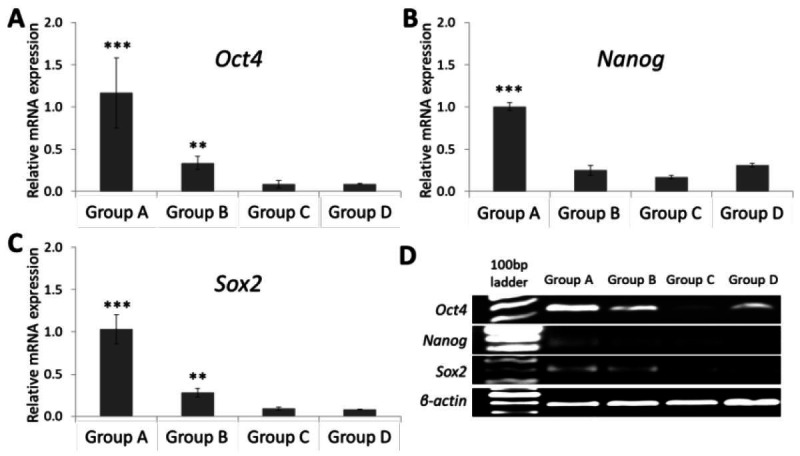
Quantitative Reverse transcriptase polymerase chain reaction analysis shows the expressions of stemness markers: (A), (B), (C) mRNA expression levels of Oct4, Nanog, and Sox2 (D) agarose gel electrophoresis and Gelred staining after qRT-PCR. ***P < 0.001 (group A) vs. other groups, **P < 0.01 (group B) vs. group C and group D.

**Figure 2:**
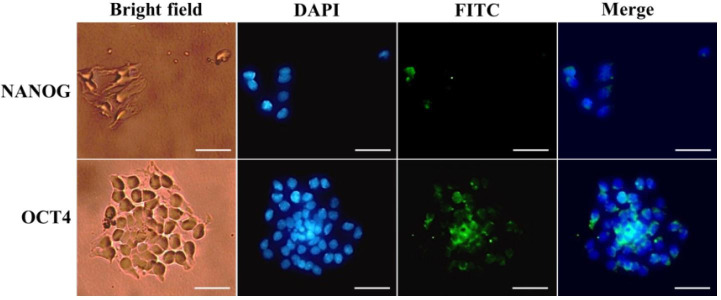
Immunofluorescence staining of GCs: OCT4 and NANOG expression were detected in fresh GCs from bovine ovarian follicle, which was smaller than 2 mm in diameter. Scale bar: 50 µm.

In this study, we assessed the stemness gene expression of GCs from bovine ovary follicles with different diameters and *in vitro* expansion. The qRT-PCR showed that the transcript expression of stemness markers (Oct4, Nanog and Sox2) were detected in fresh GCs from ovarian follicles with the specific bands in electrophoresis (Fig. 1). These stemness gene expressions were down-regulated in GCs following the increase of bovine ovary follicle diameter. The Oct4, Nanog and Sox2 transcript expression were highest in group A which was smaller than 2 mm in diameter. GCs of group B had a higher Oct4 and Sox2 transcript expressions than group C and D, but there was no significant difference in Nanog expression between these groups. qRT-PCR also revealed a higher Oct4 transcript expression than Nanog and Sox2 transcripts in group A follicles. During primary culture, the GCs attached to the culture dish in 3 hours. Immunohistochemistry showed that both OCT4 and NANOG markers were expressed in the nuclei and cytoplasm of GCs (Fig. 2).

### Stemness marker expression of GCs from in vitro expansion

**Figure 3:**
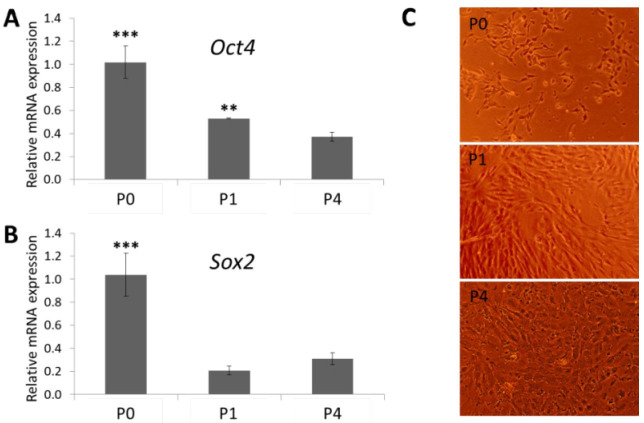
Quantitative Reverse transcriptase polymerase chain reaction analysis shows the expressions of stemness markers (A), (B) mRNA expression levels of Oct4 and Sox2 (C) granulosa cell morphology from passage 0 (P0), passage 1 (P1), and passage 4 (P4). ***P < 0.001 (P0) vs. P1 and P4, **P < 0.01 (P1) vs. P4.

The Group A follicles were used for GCs isolation and *in vitro* culture to estimate stemness marker expression. The results demonstrated that the Oct4 and Sox2 transcripts were determined in GCs in the *in vitro* expansion, however, these gene expressions were down-regulated from passage 1 to passage 4 (Fig. 3). The Oct4 transcript expression of GCs from passage 1 was higher than passage 4, but there was no significant difference in Sox2 transcript expression between the two passages.

In real time RT-PCR analysis, the Ct values were recorded when RFUs (relative fluorescence units) were higher than 100 (this is the threshold in Piko-Thermo Realtime System). The RFUs of Oct4 and Sox2 raised higher than 3500 and 1000, respectively, while all RFUs of Nanog from P0, P1, and P4 were lower than 90. Thus, there was no Nanog transcript expression of bovine GCs from P0, P1, P4.

## Discussion

Recently, the potential of ovarian somatic stem cells has been increasingly described. The ovaries have been proved to contain somatic stem cell population in ovarian epithelium layer (Parte *et al*., 2011), stromal layer and theca layer (Gong *et al*., 2010; Bui *et al*., 2014). One of the first study, which presented the potential of GCs was indicated by Kossowska-Tomaszczuk *et al*. (2009). They showed that GCs could *in vitro* differentiate into other cell types such as neurons, chondrocytes, and osteoblasts (Kossowska-Tomaszczuk *et al*., 2009).

In our study, the fresh GCs from bovine ovary follicles expressed stemness markers (Oct4, Nanog, and Sox2). This stemness transcripts of GCs were down-regulated when follicle diameter increased. All of bovine ovarian follicles in this study were tertiary follicles (antral follicles), which are about 0.5 to 25mm in diameter (Townson *et al*., 2012). The GCs are highly differentiated in the pre-ovulatory follicle in folliculogenesis (Li *et al*., 2012). It appears that differentiation is a cause of reduced expression of stemness markers. Therefore, the potential of granulosa stem-like cells could be decreased during antral stage follicular development. We also investigated the stemness marker expression during *in vitro* expansion, in which the Oct4 and Sox2 transcript expression while Nanog transcript was not expressed. These results showed that bovine GCs progressively lost their original characteristics during *in vitro* expansion.

The other studies also indicated that the stemness markers were detected in GCs in different animals. Porcine GCs from growing and luteinizing follicles showed an expression of SOX2 and NANOG markers immediately after isolation while OCT4 expression was not detected (Mattiolia *et al*., 2012). These stemness markers expressions were maintained during the *in vitro* expansion of porcine GCs (Mattiolia *et al*., 2012). Additionally, the Oct4 mRNA was not expressed in the collected follicular-phase porcine GCs (Oki *et al*., 2012). However, the expression of human GC stemness markers was different from porcine. The OCT4 was expressed in the freshly collected luteinizing GCs and remained expressed in the luteinizing GCs throughout the culturing (Dzafic *et al*., 2013). Another study also showed that subpopulation of luteinized GCs in healthy human ovarian follicles expressed Oct4 transcripts (Varras *et al*., 2012). Our results are consistent with those above studies suggesting that mammalian ovary follicles contain GC populations, which exhibit their stemness properties. Moreover, the stemness marker expressions of granulosa stem-like cells are different from species, especially Oct4 and Nanog which are the most important regulators of stemness (Boyer *et al*., 2005).
